# Targeted temperature management: a health economic perspective

**DOI:** 10.1186/1471-227X-15-S1-A5

**Published:** 2015-06-24

**Authors:** Wilhelm Behringer

**Affiliations:** 1 Department for Emergency Medicine, University Hospital Jena, Germany

## 

More and more limited financial resources in the health care sector call for an economic evaluation of potential new therapies. The economic evaluation of a new therapy is complex and depends not only on the perspective of the various stakeholders, but also on the country-specific characteristics of various reimbursement systems. From the patient perspective, the costs for new therapies might not be a concern, as long as the insurance system covers the costs. From the hospital perspective, a new therapy which reduces the length of stay might be worthwhile in a DRG-based reimbursement system, but not in a pay-per-patient-day reimbursement system. To respect the perspective of the society, a cost-effectiveness analysis could be expressed in terms of a ratio where the denominator is a gain in quality-adjusted life years (QALY) and the numerator is the cost associated with the health gain.

QALY is a measure of disease burden and takes into account the quality of life measured as the Health Utility Index (HUI, dead = 0 and perfect health = 1) and quantity of life lived measured as survival time: QALY = survival time × HUI (for example, 4 years survival with HUI 0.5 = 2 QALY, or 2 years survival with HUI 1 = 2 QALY).

Previous studies reported that mild therapeutic hypothermia improves neurologic recovery and survival in patients resuscitated from cardiac arrest. The estimated number needed to treat is only six patients; nevertheless, target temperature management has hardly been implemented in post-cardiac arrest treatment. The aim of the current study was to evaluate the cost-effectiveness of therapeutic hypothermia in post-cardiac arrest patients. The QALY–cost ratio was chosen as the primary outcome.

To assess the cost-effectiveness of temperature management, data from a Cochrane review about mild hypothermia after cardiac arrest including 398 patients were analyzed. The QALY–cost ratio was calculated based on the cerebral performance score and total treatment costs after cardiac arrest. The assumed costs included potential cooling procedure, hospital stay, rehabilitation, and potential defibrillation implantation in Austria.

Total treatment costs per 100 patients were €4.1 million for patients treated with mild hypothermia, and €3.7 million for patients with standard care. Post-arrest patients receiving mild hypothermia gained an average of 1.11 QALY compared with conventional care. This resulted in a cost-effectiveness ratio of €3.827 per QALY.

Targeted temperature management at 32 to 34°C after cardiac arrest seems to be a very cost-effective treatment, with €3.827 per QALY far below commonly used benchmarks for assessing the cost-effectiveness of an intervention. These results may provide an economic rationale for implementing targeted temperature management as a standard treatment after cardiac arrest.

